# Spin–orbit torques in normal metal/Nb/ferromagnet heterostructures

**DOI:** 10.1038/s41598-021-99745-4

**Published:** 2021-10-26

**Authors:** Min Hyeok Lee, Gyungchoon Go, Yong Jin Kim, In Ho Cha, Gyu Won Kim, Taehyun Kim, Kyung-Jin Lee, Young Keun Kim

**Affiliations:** 1 Department of Materials Science and Engineering, Korea University, Seoul, 02841 Republic of Korea; 2 Department of Physics, Korea Advanced Institute of Science and Technology, Daejeon, 34141 Republic of Korea; 3 KU-KIST Graduate School of Converging Science and Technology, Korea University, Seoul, 02841 Republic of Korea

**Keywords:** Materials science, Physics

## Abstract

Quantifying the spin–orbit torque (SOT) efficiency with changing the layer thickness is crucial for understanding the physical background of SOT. This study investigates the Nb-thickness-dependent SOT efficiency of two types of layered heterostructures: Ta/Nb/CoFeB and Pt/Nb/CoFeB. We find that the Nb thickness dependence of the SOT efficiency in the two samples is quite different. In the Pt/Nb series, the SOT sign changes according to the thickness variation because Pt and Nb have different spin–orbit coupling signs. We observe the resulting reversal in switching polarity through current-induced SOT switching experiments. However, due to the same spin–orbit coupling signs of Ta and Nb, no such polarity reversal was observed in Ta/Nb series. Further, we extract the spin diffusion length of Nb in each heterostructure. These results provide a systematic understanding of the material- and thickness-dependent SOT characteristics.

## Introduction

When an in-plane current is applied to a normal metal (NM)/ferromagnet (FM) heterostructure, a spin current generated in the NM flows to the FM and exerts torque. This torque, known as the spin–orbit torque (SOT), can manipulate the magnetization of the FM^1–3^. The SOT enables a more rapid and energy-efficient operation than conventional spintronic applications based on spin–transfer torque^4^. Thus, the SOT is considered key to the development of future magnetic memory devices. The generation of spin current is a result of the spin Hall effect (SHE)^2,5–9^ and the Rashba–Edelstein effect (REE)^1,3,10–13^, which arise from spin–orbit coupling (SOC). The SHE is a result of the bulk SOC effect. When an unpolarized electric current flows through the material with strong SOC, the SOC-induced scattering causes the down spin and up spin to flow in opposite directions. The mechanism generates a pure spin current in a transverse direction to the electric current. The REE originates from an interface with broken inversion symmetry, which induces an effective electric field in the system. Consequently, a spin accumulation is induced at the interface. The SOT induced by both mechanisms can be divided into two vector components: a damping-like torque (DLT, ${{\varvec{\tau}}}_{DLT}= \widehat{{\varvec{m}}}\times (\widehat{{\varvec{m}}}\times \widehat{{\varvec{s}}}))$ playing a critical role in magnetization reversal and field-like torque (FLT, ${{\varvec{\tau}}}_{FLT}= \widehat{{\varvec{m}}}\times \widehat{{\varvec{s}}}$, where $\widehat{{\varvec{m}}}$ and $\widehat{{\varvec{s}}}$ are unit vectors of magnetization and spin polarization, respectively) assisting the switching process^14–19^.

In the SOT experiment, the NM thickness variation is typically employed to investigate spin transport characteristics, critical for understanding the SOT mechanisms. Most SOT studies have considered the simple NM/FM bilayer structures^3,9–11,15–23^. In these cases, the NM layer (or NM/FM interface) is considered a single spin current (spin torque) source, and the spin current is absorbed into the magnetization of the FM layer. Recently, either NM1/NM2^17,19,24,25^ or FM/NM^26–32^ bilayer structures have been used as a spin current source to enhance the SOT efficiency. For understanding the underlying SOT mechanism in trilayer structures, it is crucial to carry out thickness-dependence SOT measurements.

This study experimentally investigates the NM1/NM2/FM trilayer structures where NM1 is either Pt or Ta and NM2 is Nb. We carry out SOT measurements with varying the Nb thickness. In these NM/Nb/FM structures, the Nb layer plays the role of spin transport and spin current source. As Nb is known to exhibit weak SOC compared to Ta and Pt^33,34^, we chose Nb for NM1 in this study. We expect to observe the thickness-dependent transport characteristic of the spin current more efficiently when choosing Nb as a middle layer in trilayer structures. The SOT efficiency in the Nb/CoFeB system was approximately − 0.0298 ± 0.00035; this demonstrated the weak SOC effect of Nb. We investigate two types of NM/Nb/CoFeB trilayer in which the NM was either Ta or Pt. According to several previous studies^2,15,16,20–23^, Ta and Pt both showed higher SOT efficiencies than Nb but had opposite signs of SOC with each other. The SOT value increased and saturated with increasing the Nb thickness in both series through the bulk spin diffusion model^35,36^. Varying the Nb thickness would enable the precise switching-polarity reverse in Pt/Nb/FM structure when it changed around critical thickness. In contrast, there would be no such reverse in the Ta/Nb/FM structure. We confirm this polarity reversal behavior by both second harmonics and current-induced SOT switching measurements, suggesting well-designed systematical observation of the thickness dependence of SOT.

## Results

### Magnetic properties and spin–orbit torque of Nb/CoFeB/MgO/Ta

We analyzed the magnetic properties of Nb (${t}_{Nb}$)/CoFeB (0.9)/MgO (1)/Ta (2) (in nm) films (${t}_{Nb}$ = 3, 5, 7, 9, and 15 nm). The magnetic hysteresis loops measured by vibrating sample magnetometry (VSM) demonstrated that all samples exhibited well-defined perpendicular magnetic anisotropy (PMA), except for the one with a 3 nm thick Nb layer (Fig. S1 in Supplementary Note 1).

**Figure 1:**
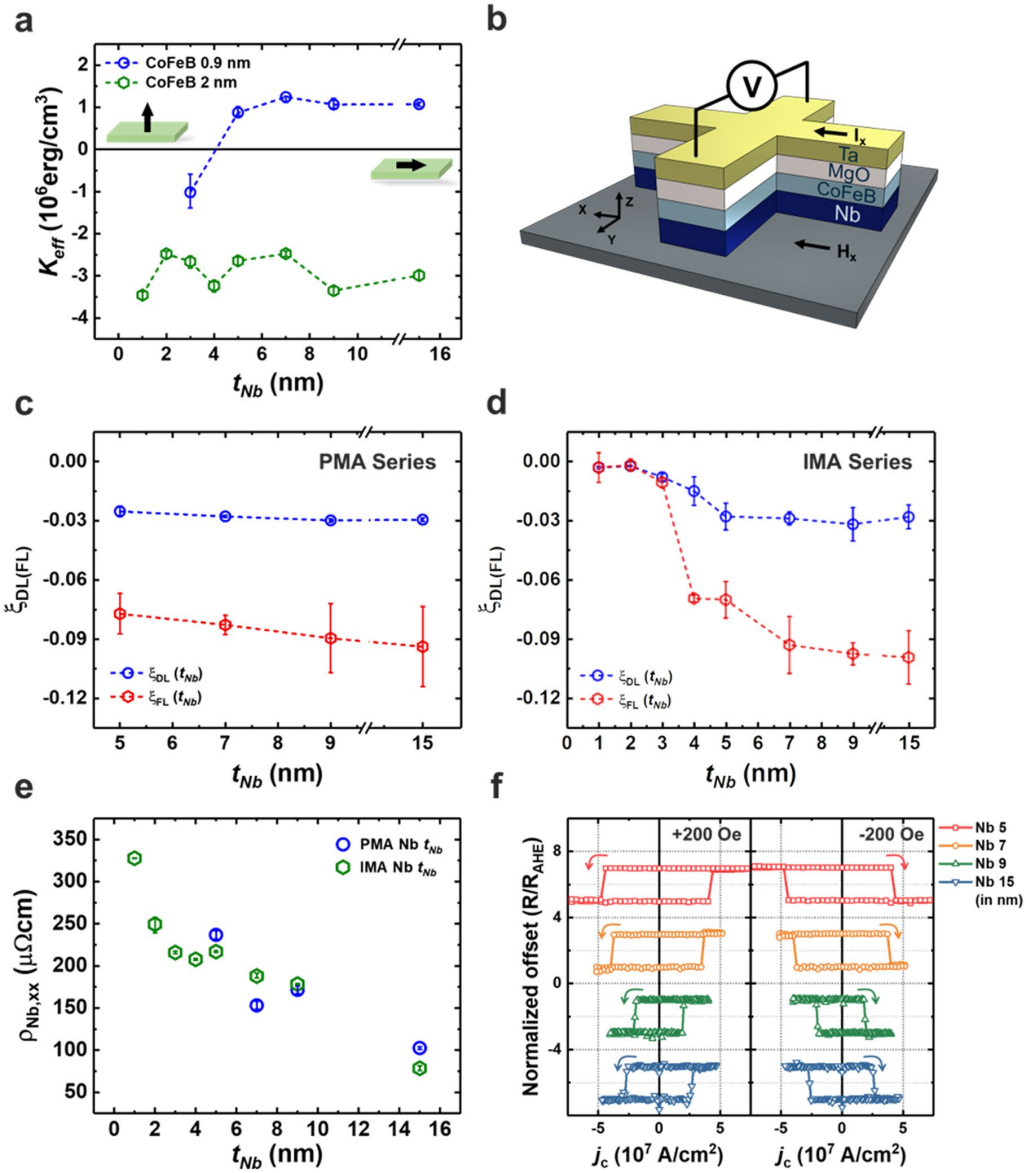
Magnetic and SOT properties for the Nb/CoFeB/MgO/Ta films. (**a**) Values of effective anisotropy energy *K*_*eff*_ as a function of ${t}_{Nb}$. The inset is the schematic of the easy axis of the CoFeB layer. (**b**) Schematic of the Hall bar structure to measure spin Hall properties. (**c**) ${\xi }_{DL}^{Nb}$ and ${\xi }_{FL}^{Nb}$ as a function of ${t}_{Nb}$ for each with varying Nb thickness in the PMA samples. (**d**) ${\xi }_{DL}^{Nb}$ and ${\xi }_{FL}^{Nb}$ as a function of ${t}_{Nb}$ using film structures with 2 nm thick CoFeB layers exhibiting IMA. (**e**) Resistivity of Nb layer as a function of ${t}_{Nb}$ in PMA (blue points) and IMA structures (green points), respectively. (**f**) Current induced-SOT switching curve of Nb/CoFeB/MgO/Ta devices.

Effective magnetic anisotropy energy (${K}_{u,eff}$) of approximately ~ 1 Merg/cm^3^ (Fig. 1a) was quantified based on the integral area difference of the M–H loops between the hard axis and easy axis. As ${t}_{Nb}$ increases by more than 5 nm, ${K}_{u,eff}$ saturates; this signifies well-developed and constant magnetic anisotropy regardless of Nb layer thickness.

Samples that exhibited PMA were selected to fabricate a Hall bar device to measure SOT. Figure 1b presents the geometry of the harmonics measurement^37,38^. Figure 1c illustrates the SOT efficiency (${\xi }_{DL/FL}$) of Nb as a function of ${t}_{Nb}$:

1 $${\xi }_{DL/FL}=\frac{2e}{\mathrm{\hslash }}\frac{{M}_{s}{t}_{F}{B}_{DL/FL}}{{j}_{c}}$$

where ${B}_{DL/FL}$ is an effective damping-like (DL)/field-like (FL) spin–orbit field^21,23^, ${t}_{F}$ is the thickness of FM, and ${j}_{c}$ is the charge current density. We analyzed the first and second harmonic signals as a function of the magnitude of the external field (Fig. S2 in Supplementary Note 2). The largest ${\xi }_{DL}$ value in the Nb/CoFeB heterostructure was − 0.0298 ± 0.000839, corresponding to the device with a 9 nm thick Nb layer. There was no thickness dependence on ${\xi }_{DL}$ when the Nb layer thickness had increased from 5 to 15 nm, in contrast with its low DLT efficiency, Nb exhibited a relatively high FLT efficiency of up to − 0.0937 ± 0.0203. Furthermore, given the large error bars, the FLT appeared to lack thickness dependence within the tested Nb thickness range.

According to the spin diffusion model, ${\xi }_{DL(FL)}$ should increase with the thickness of the spin current source layer and then saturate when the thickness of the spin current source layer exceeds its spin diffusion length. However, we cannot see the thickness dependence of ${\xi }_{DL}$, neither of ${\xi }_{FL}$, as PMA was not exhibited when ${t}_{Nb}<5$ nm. Thus, we fabricated another series that consist of Nb (${t}_{Nb}$)/CoFeB (2)/MgO (1)/Ta (2) (in nm) structure (${t}_{Nb}$ = 1, 2, 3, 4, 5, 7, 9, and 15 nm). These sample series possess in-plane magnetic anisotropy (IMA) because we increased the CoFeB layer thickness to 2 nm. Figure 1b shows that the ${K}_{u,eff}$ of the films with 2 nm thick CoFeB layer is roughly constant with some fluctuations. We measured SOT properties with the harmonics using the in-plane measurement method (Supplementary Note 3). Figure 1d shows the thickness dependence of ${\xi }_{DL(FL)}$. In this case, ${\xi }_{DL(FL)}$ showed increasing and saturation behavior when the layer thickness exceeded a certain value. Although ${\xi }_{DL(FL)}$ was measured in different geometry, the saturated values of IMA films were comparable with those of PMA ones. We considered the current shunting effect when extracting ${\xi }_{DL(FL)}$ by subtracting the amount of current flowing through the FM layer. We obtained the electrical resistivity of the Nb using the parallel circuit model. This calculation assumed that current only flowed through the Nb and CoFeB layers, not the highly resistive MgO and Ta capping layers. We can confirm that the thickness and resistivity were inversely proportional to each other, as shown in Fig. 1e. The ${\rho }_{xx}^{Nb}$ obtained from PMA and IMA films were comparable with each other.

The efficiency obtained in our SOT measurements (${\xi }_{DL}=-0.0298$) differed from those in a spin-pumping measurement with the Nb/Ni_80_Fe_20_ heterostructure (${\xi }_{DL}=-0.001$)^39^ and in the spin absorption measurement in the lateral spin-valve structure involving the Ni_80_Fe_20_/Cu/Nb junction (${\xi }_{DL}=-0.009$)^40^. Although the efficiency of the charge-to-spin conversion in this study was higher than that obtained in previous studies employing other measurement methods and material combinations, it was evident that the magnitude of SOT was still low compared to that of other 5d heavy metals such as Pt (~ 0.09)^20–23^, Ta (~ − 0.12)^2,15,16,19^, and β–W (~ − 0.33)^18,19,41^. We also conducted the current-induced SOT switching measurements using the samples with PMA to verify that the relatively low efficiency is sufficient to switch the FM layer effectively. We patterned the device using ion milling to fabricate a dot-shaped structure of the CoFeB/MgO/Ta layers with a 4 μm diameter to measure the SOT switching. Figure 1f shows clear SOT switching in Nb/CoFeB heterostructures. When the external field was induced along the + x axis, the FM showed counterclockwise switching behavior. Under the external field along the − x axis, we can observe the reversal of the switching polarity, which is the representative evidence of SOT-induced magnetization switching.

We designed a trilayer structure based on the above experiments about the spin torque properties of Nb to further discern thickness-dependent SOT behavior. We investigated NM (Ta or Pt)/Nb/CoFeB heterostructures where the primary role of Nb is as a diffusion layer, not a spin current source; that is, a spin current originally generated in Ta or Pt diffuses and reaches the CoFeB layer. By conducting SOT measurement in these trilayer structures, we can systematically observe the thickness-dependence of spin transport properties.

### Analysis of thickness dependence of SOT in NM/Nb/FM tri-layer

In the previous section, we demonstrated that the ${\xi }_{DL}$ value of Nb was considerably small compared to that of Ta (~ − 0.12) or Pt (~ − 0.09). Thus, the combination of an NM (Ta or Pt) with Nb was advantageous for observing how the spin current transported with varying Nb thickness. As the spin current generated in Ta (or Pt) is greater than that generated in Nb or Nb/FM, the SOT measured in this structure was primarily determined by how the spin current generated initially in Ta (Pt) diffused through Nb. The Ta (Pt) layer was also selected owing to an additional advantage. Each material exhibits a particular sign corresponding to the SHE, which determines the sign of the SOT. For example, Ta and W exhibit a SHE with a negative sign, whereas Pt shows a positive sign^16,17^. As described in the previous section, Nb exhibits a SHE with a negative sign, similar to Ta and in contrast with Pt. Note that in the Ta/Nb structure, the spin currents generated in Ta and Nb were constructively added. In contrast, in the Pt/Nb structure, the spin current generated in Pt and Nb were destructively added.

We deposited films consisting of Ta (3)/Nb (${t}_{Nb}$)/CoFeB (0.9)/MgO (1)/Ta (2) and Pt (3)/Nb (${t}_{Nb}$)/CoFeB (1.1)/MgO (1)/Ta (2) (in nm) and measured the magnetic anisotropy using VSM. We employed a thin Nb layer in the Pt/Nb series to observe the effect of the spin current from Pt; this competed with that of Nb. Thus, ${t}_{Nb}$ varied as 3, 5, 7, 9, and 15 nm for the Ta/Nb series and as 0, 1, 2, 3, 4, 5, 7, 9, and 12 nm for the Pt/Nb series. PMA was well-developed when ${t}_{Nb}$ ≥ 5 nm for Ta/Nb, whereas it was confirmed when ${t}_{Nb}$ ≥ 1 nm for Pt/Nb (Fig. S1 in Supplementary Note 1).

**Figure 2:**
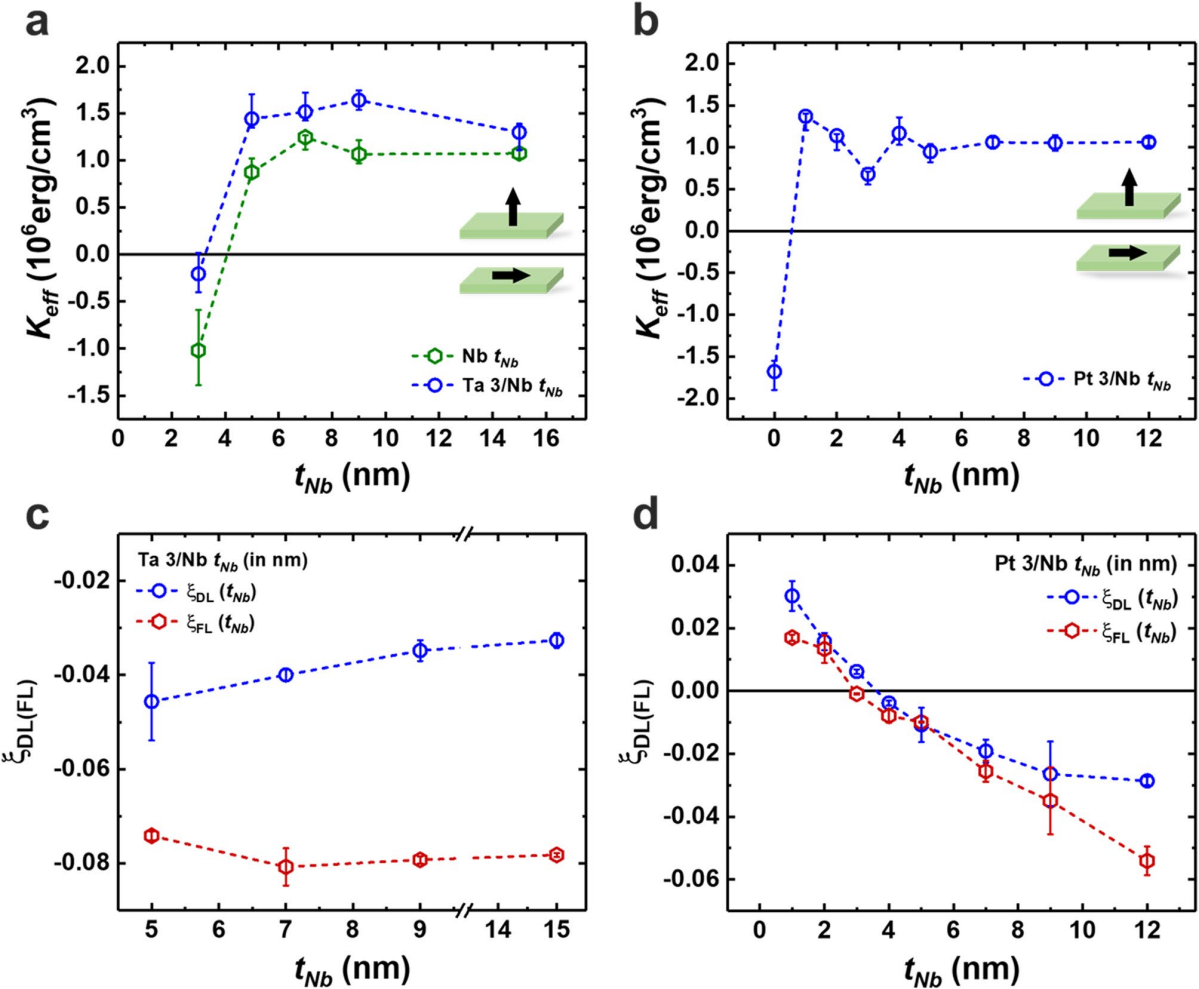
Magnetic and SOT properties for the Ta or Pt/Nb/CoFeB/MgO/Ta films. (**a**, **c**) Effective anisotropy energy *K*_*eff*_ and ${\xi }_{DL(FL)}$ as a function of ${t}_{Nb}$ in the Ta/Nb series. The green dots in a show the *K*_*eff*_ of Nb/CoFeB/MgO/Ta for comparison. (**b**, **d**) Effective anisotropy energy *K*_*eff*_ and ${\xi }_{DL(FL)}$ as a function of ${t}_{Nb}$ in the Pt/Nb series, respectively.

Figure 2a,b illustrate the magnetic anisotropy energy of the films in the Ta/Nb and Pt/Nb series, respectively. The ${K}_{u,eff}$ value of the Ta (3)/Nb (3)/CoFeB (0.9)/MgO (1)/Ta (2) film was negligible, although it had increased to ~ 1.5 Merg/cm^3^ as ${t}_{Nb}$ exceeded 3 nm. Compared to Nb, the overall ${K}_{u,eff}$ value increased and showed the same trend. The enhancement in ${K}_{u,eff}$ value originates from differences in the Nb/CoFeB interface quality^42,43^. The crystal structure and atomic radius of Nb were the same as those of Ta; this implies that the Ta layer introduced under the Nb layer played a role as the seed layer. For Pt/Nb in Fig. 2b, the PMA was well-defined when Pt was introduced under the Nb layer. Further, ${K}_{u,eff}$ value was comparable with that of Nb/CoFeB/MgO/Ta, as shown in Fig. 1b.

All PMA films were fabricated into Hall bar devices, and harmonics were measured using these devices. The first and second harmonic signals of the two series, Ta/Nb and Pt/Nb, are shown in Fig. S2 of Supplementary Note 2. Again, the SOT efficiency was calculated by considering the current shunting effect. We fabricated two reference films consisting of Ta (3) or Pt (3)/CoFeB (0.9)/MgO (1)/Ta (2) to obtain ${\rho }_{xx}^{Nb}$ corresponding to each thickness which was calculated by deducting the ${\rho }_{xx}$ value of each reference from the resistivity of the entire film (Supplementary Note 4). Figure 2c shows the ${\xi }_{DL}$ of the Ta/Nb series films as a function of ${t}_{Nb}$. Note that ${\xi }_{DL}$ enhanced to − 0.046 ± 0.0083 following the introduction of the Ta layer under the 5 nm thick Nb layer, and a further increase in ${t}_{Nb}$ gradually decreased ${\xi }_{DL}$. This decrease in ${\xi }_{DL}$ for increasing ${t}_{Nb}$ indicates that a spin current generated in the Ta layer exerted a torque on the CoFeB layer. Additionally, the amount of spin current reaching the CoFeB layer decreased due to the spin diffusion process through the Nb layer. ${\xi }_{FL}$ showed a different behavior from ${\xi }_{DL}$. Within the range of ${5 \le t}_{Nb}\le 15$ nm, ${\xi }_{FL}$ was already saturated and showed a value of about − 0.08. This behavior and the magnitude of ${\xi }_{FL}$ were comparable with the thickness-dependent ${\xi }_{FL}$ in Nb/CoFeB bilayer structure shown in Fig. 1c. In the Pt/Nb series, ${\xi }_{DL}$ changes its sign as ${t}_{Nb}$ varies near 3 nm, as shown in Fig. 2d. Films with ${t}_{Nb}$ values of 1 and 2 nm exhibited a positive ${\xi }_{DL}$, whereas those with ${t}_{Nb}$ thicknesses over the 3 nm had a negative ${\xi }_{DL}$. The sign reversal of the SOT ratio in Pt/Nb/CoFeB can be understood by the following argument. When Nb is relatively thin, the spin current generated in Pt mainly contributes to the SOT. When the Nb thickness was increased to 3 nm, the SOT contributed by Pt and Nb at the interface may cancel out. As the Nb thickness continued to grow, less or even no spin current generated in Pt could reach the Nb/CoFeB interface, where the contribution from the spin current generated in Nb led to the sign reversal of SOT. We also observed similar SOT sign reversal in ${\xi }_{FL}$ and the magnitude of ${\xi }_{FL}$ increased to about − 0.054, a value that was not yet saturated.

**Figure 3:**
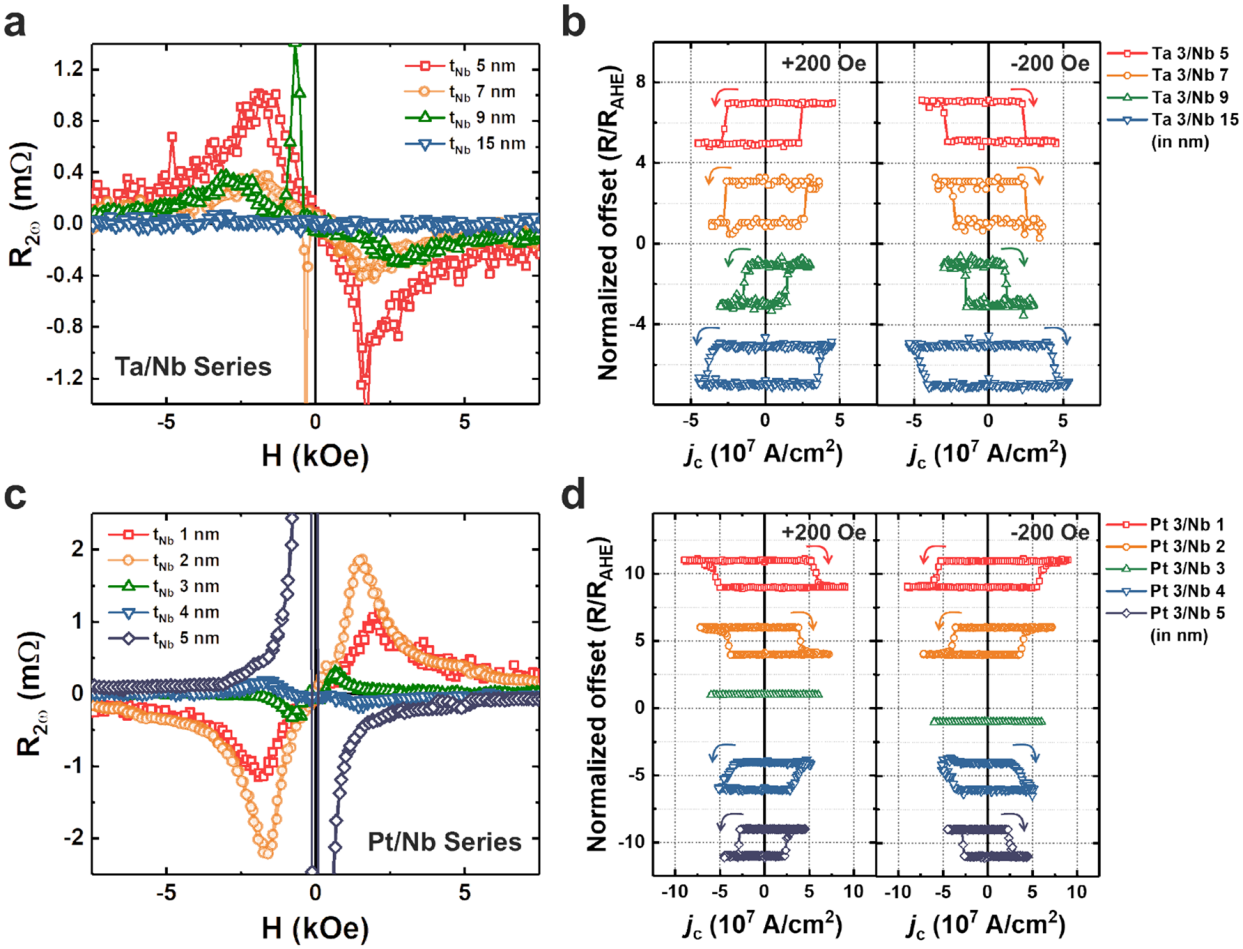
Comparison of the polarity of the second harmonic signal and current-induced SOT switching. (**a**, **c**) Second harmonic signal of the Ta/Nb (**a**) and Pt/Nb series (**c**), respectively. The sign change occurs when the thickness of Nb exceeds 3 nm in Pt/Nb, but there was no such change in Ta/Nb series. (**b**, **d**) Current induced-SOT switching curve of the Ta/Nb (**b**) and Pt/Nb series (**d**).

Figure 3a,c show the second harmonic responses of the Ta/Nb and Pt/Nb series, respectively, which used to extract the ${\xi }_{DL}$ as shown in Fig. 2c,d. We can obtain the second harmonic signal in the Ta/Nb series with the same contour as the materials with negative SOC signs. However, the situation in the Pt/Nb series was different. In Fig. 3c, a positive signal was obtained for films with ${t}_{Nb}$ ≤ 3 nm. If ${t}_{Nb}$ > 3 nm, the harmonic responses showed a negative signal. In addition, we conducted the current-induced SOT switching measurements to verify the sign of ${\xi }_{DL}$ independently, as shown in Fig. 3b,d. We induced the electrical current to a dot-shaped device to measure SOT switching. When the electrical current was induced to the device under an external field parallel to the current direction (+ x), the magnetization of the FM layer rotated counterclockwise for the Ta/Nb devices within all Nb thickness range. This result showed the same sign as the harmonics experiment. The switching polarity change according to the direction of the external magnetic field confirmed the SOT-induced switching. In the Pt/Nb series, the magnetization rotated clockwise for films with ${t}_{Nb}$ of 1 and 2 nm under the + 200 Oe external field. However, magnetization rotated counterclockwise for films with ${t}_{Nb}$ of 4 and 5 nm. No SOT switching was observed in the sample with ${t}_{Nb}$ of 3 nm because of its low ${\xi }_{DL}$ (0.0016 ± 0.0011). The results show that for cases with a thin Nb, the spin current generated in the Pt layer dominates the current generated in the Nb or at the Nb/CoFeB interface.

### Microstructural analysis of Nb layers

**Figure 4:**
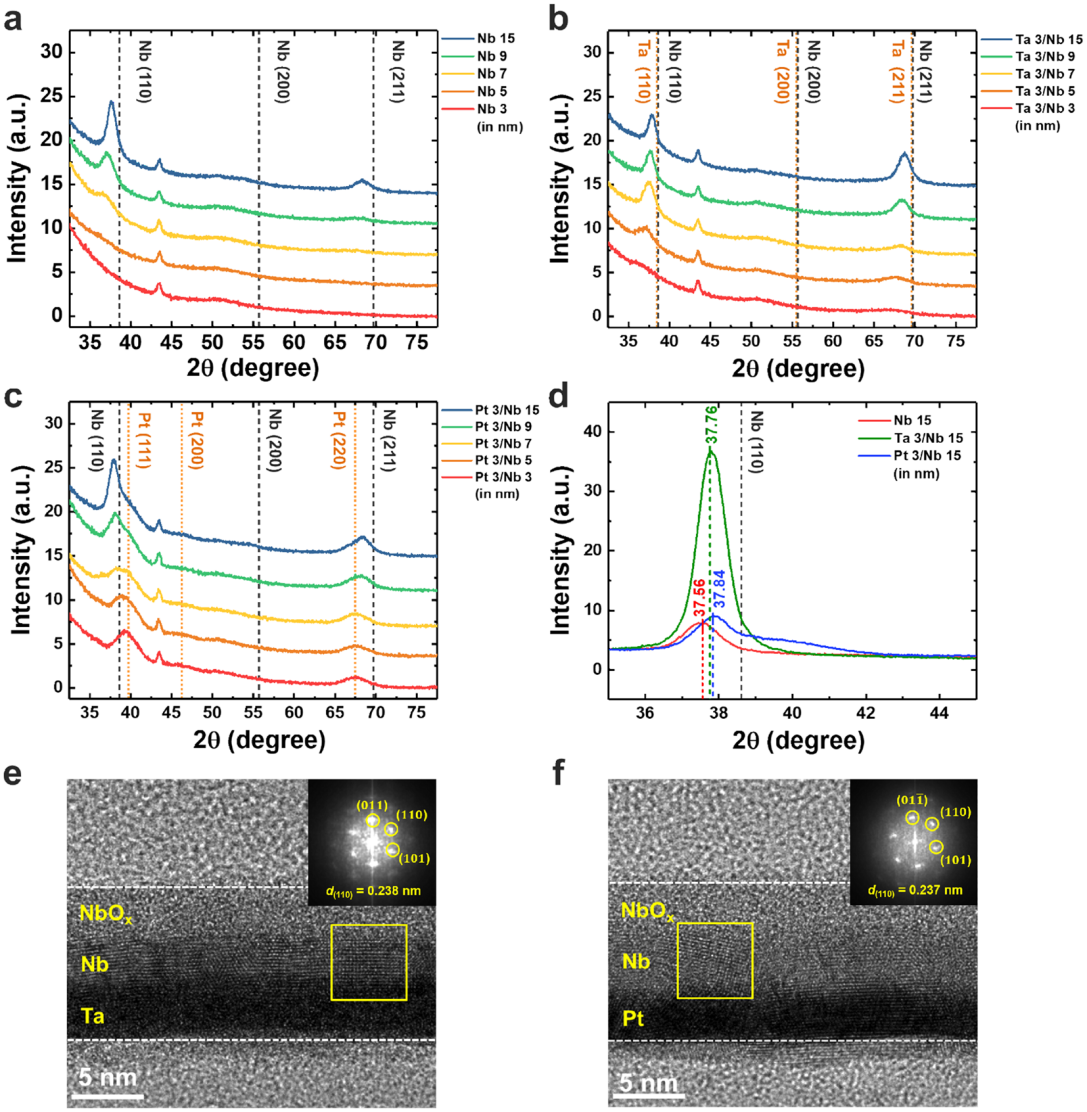
XRD and TEM analysis of Nb thin films with the different buffer layers. (**a**–**c**) Peak signals of Nb thin films as a function of ${t}_{Nb}$ in (**a**) Si/SiO_2_/Nb ${t}_{Nb}$, (**b**) Si/SiO_2_/Ta 3/Nb ${t}_{Nb}$ and (**c**) Si/SiO_2_/Pt 3/Nb ${t}_{Nb}$ (${t}_{Nb}$ = 3, 5, 7, 9, 15 nm) films, respectively. (**d**) Bragg mode scan with the angle range from 35° to 45° of the samples with the 15 nm of Nb layer. (**e**, **f**) TEM analysis of Ta 3/Nb 5 nm (**e**) and Pt 3/Nb 5 nm films (**f**) following annealing at 300 °C for 1 h. The inset of each figure shows the SAED image of Nb on the Ta and Pt underlayers.

Several studies have suggested that SOT efficiency changed according to the different crystal phases of NM materials^44,45^. Nb thin films were grown on the three different underlayers (SiO_2_, Ta, and Pt). To check the Nb crystallinity effect on the result of SOT experiments above, we fabricated three series of samples; (1) Si/SiO_2_/Nb ${t}_{Nb}$, (2) Si/SiO_2_/Ta 3/Nb ${t}_{Nb}$, and (3) Si/SiO_2_/Pt 3/Nb ${t}_{Nb}$ (${t}_{Nb}$ = 3, 5, 7, 9, 15 nm). We measured the crystallinity of Nb in each series using X-ray diffraction (XRD). We first performed a grazing incidence mode scan to observe the peak signal of few nanometer-thick films as shown in Fig. 4a–c.

On the SiO_2_ underlayer, the (110) peak of body-centered cubic (bcc) Nb begins to appear when ${t}_{Nb}$ was 7 nm or more. We observed this trend in the Pt series (see Fig. 4c), but the peak could be observed from the thinner 5 nm Nb film in the Ta series. This result showed that the Ta layer well played a role as the seed layer. However, peaks of Nb slightly shifted to the lower angle from the reference peak in all cases. Therefore, we scanned the thickest thin film (${t}_{Nb}$ = 15 nm) of each series using the normal Bragg mode with the angle range from 35° to 45° as shown in Fig. 4d. In all cases, the Nb (110) peak shifted slightly to the lower angle, and their peak positions were 37.56° (SiO_2_), 37.76° (Ta), and 37.84° (Pt underlayer), respectively. There was just a neglectable difference between the films with different under layers. The peak intensity of the Ta case was seven times higher than the other two cases, which was a consistent result with the lower resistivity of the Nb layers on the Ta layer compared with those of on the SiO_2_ and Pt (Supplementary Note 4). Through this Bragg mode result, the peak near 43° seen in the grazing incidence mode scan was identified as the holder peak, not the peak of the sample.

Figure 4e,f show the cross-sectional transmission electron microscopy (TEM) images of Ta 3/Nb 5 nm and Pt 3/Nb 5 nm, respectively. The absence of a barrier layer over the Nb layer prompted the oxidation of the top region, as shown in the amorphic and brighter parts. The Nb layer, approximately 3 nm thick, remained unoxidized and appeared to have good crystallinity in both structures. Each inset shows the selected area electron diffraction (SAED) pattern of Nb. Regardless of underlayers, Nb exhibited bcc structure with spacing *d*_(110)_ = 0.238 and 0.237 nm, respectively. These values are comparable with those from XRD analysis (*d*_(110)_ = 0.2374 nm in Ta/Nb and 0.2370 nm in Pt/Nb) obtained by using the Bragg equation. The XRD and TEM analysis suggests that the underlayer type has a negligible effect on the microstructure of the Nb films.

### Diffusive behavior of the spin current in the Nb layer

**Figure 5:**
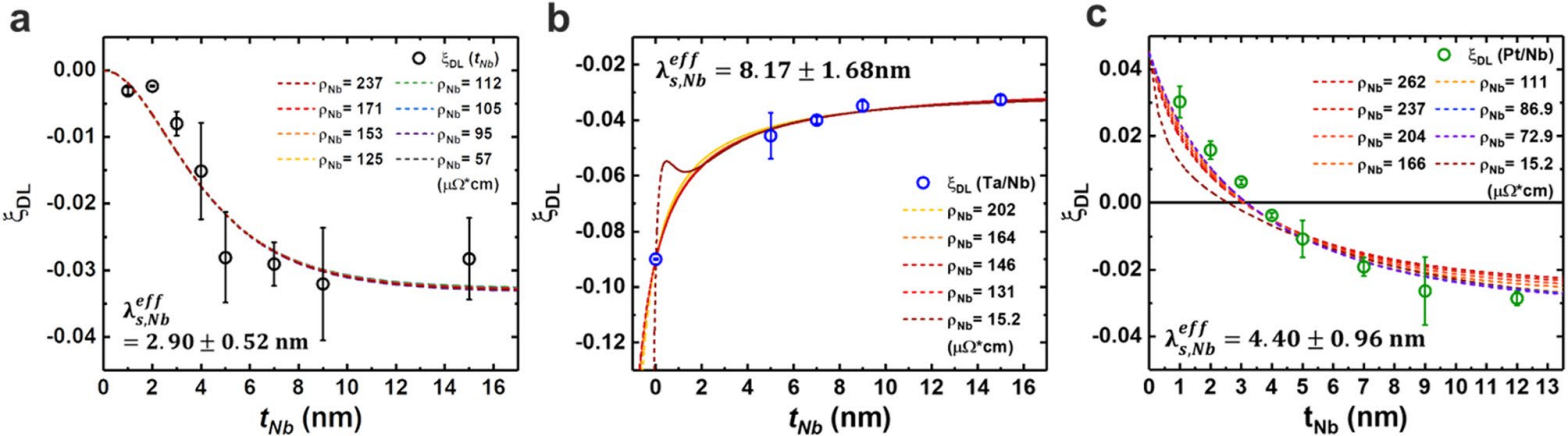
Extraction of the spin diffusion length. (**a**–**c**) ${\xi }_{DL}$ as a function of ${t}_{Nb}$ in the Nb/CoFeB heterostructures, Ta/Nb/CoFeB and Pt/Nb/CoFeB tri-layers, respectively. Each dotted line shows the fitting using the drift–diffusion equation with different resistivities of Nb.

We further carried out a theoretical analysis of the experimental results for Nb/CoFeB bi-layer and NM/Nb/CoFeB tri-layers using the spin drift–diffusion model (Supplementary Note 5). We fit experimental data to the equation derived via the drift–diffusion model (see Fig. 5). The resistivity of the Nb layer ${\rho }_{xx}^{Nb}$ was a variable dependent on ${t}_{Nb}$, as described in Fig. 1e and Supplementary Note 4. We put the ${\rho }_{xx}^{Nb}$ values in the drift–diffusion equation and fit the data for each ${\rho }_{xx}^{Nb}$ value. The bulk resistivity of Nb (15.2 μΩ cm) was also considered a fitting parameter that corresponds to the fitting line; further, this exhibited the most significant deviation from data points. However, the resistivity variation of Nb thin films in this study did not affect the fitting trend.

The spin diffusion length of the Nb extracted from the ${\xi }_{DL}$ of Nb/CoFeB bilayers exhibiting IMA was ${\lambda }_{s, Nb}^{eff}$ = 2.90 ± 0.52 nm. However, the values from trilayer system were substantially different from what was obtained from the Nb/CoFeB structures. We obtained ${\lambda }_{s, Nb}^{eff}$ = 8.17 ± 1.68 nm from the Ta/Nb series and ${\lambda }_{s, Nb}^{eff}$ = 4.40 ± 0.96 nm from the Pt/Nb series, respectively. The difference in ${\lambda }_{s, Nb}^{eff}$ may be attributed to the model not accounting for NM/FM interfacial spin current generation. To check this, we also fitted the data using an extended spin diffusion model including the additional spin current generated by the interfacial spin–orbit coupling^46,47^ and obtained a similar value of ${\lambda }_{s}^{eff}$ with the conventional bulk model (see Supplementary Notes 5, 6). Fitting using an extended model suggests that interfacial spin current generation at the Nb/CoFeB was not dominant in our systems. Another potential cause for different values in each series is the NM (Pt or Ta)/Nb interface being another spin current source. The variations in the NM/Nb interface quality should exist depending on the different material combinations. We confirmed these variations through TEM energy dispersive spectroscopy (EDS) analysis of the Ta (or Pt)/NM heterostructure structure (Supplementary Note 7) and atomic force microscopy (AFM) analysis (Supplementary Note 8).

However, if the NM/Nb interface is a delta-function-like spin current source, this cannot explain the different ${\lambda }_{s, Nb}^{eff}$ values obtained from the Ta/Nb and Pt/Nb series. This is because a delta-function-like spin current source can only affect the magnitude of the spin current source term and does not affect the spin diffusion process through the bulk Nb layer, i.e., the spin diffusion length is not relevant to the existence of the additional spin current source but determined by an exponential decay of the spin polarization. Therefore, if the interfacial SOC is the cause of such different values, the spin current generation at the interface would have a finite length scale, depending on the material combinations. However, as such a model has not yet been established, further analysis of these experimental results with an improved model is required in future research.

## Discussion

This study systematically investigated the spin transport in Nb by measuring the SOT efficiency of Nb/CoFeB heterostructure and NM (Ta or Pt)/Nb/CoFeB tri-layers with varying Nb thicknesses. The previous studies about spin-torque efficiency of Nb investigated the properties based on other Nb/FM combinations, which showed IMA^39,40^. It is crucial to analyze the device characteristics in a structure where CoFeB, a representative candidate material for a magnetic tunnel junction, has PMA to apply spintronics devices. Therefore, we focused on Nb/CoFeB structures that exhibited PMA or IMA, then characterized Nb-thickness-dependent SOT properties. ${\xi }_{DL}$ increased as the ${t}_{Nb}$ increased and saturated to − 0.0298 ± 0.000839. Thus, we confirmed that Nb has relatively low SOT compared to 5d heavy metals, but enough to manifest the effective current-induced SOT switching.

For a more systematic experiment, we introduced two types of NM layers placed under Nb. We observed apparent thickness-dependent spin transport properties as varying with ${t}_{Nb}$. ${\xi }_{DL}$ had enhanced following the introduction of the Ta layer and gradually decreased with an increase in ${t}_{Nb}$. However, in Pt/Nb, the sign of ${\xi }_{DL}$ was opposite compared to that of Nb/CoFeB when ${t}_{Nb}\le 3$ nm, while it showed the same sign when ${t}_{Nb} >3$ nm. The current-induced switching measurement also revealed this sign reversal. We further analyze SOT results with the bulk spin diffusion model. In bilayer, similar to the spin diffusion length of Pt (1–3 nm) in earlier studies^20–23^, extracted length parameter of Nb was substantially smaller than the mean free path of Nb (~ 20 nm^48,49^), and this result violates the model assumptions. The larger ${\lambda }_{s}^{eff}$ compared to that in bilayer were obtained in trilayer structures, suggesting that the NM/Nb interfaces play a role of a spin current source, but not delta-function-like. Furthermore, it indicates that the contribution from the NM/Nb interface to the SOT may be non-negligible; this is consistent with experimental observations of the REE at the interface between two different NMs^50,51^.

## Methods

### Sample preparation

We deposited all samples using direct current (DC) and radio frequency (RF) magnetron sputtering for metals and oxides, respectively, onto 300 nm thick thermally oxidized Si wafers under a base pressure below 5 × 10^−9^ Torr. The reference film stacks consist of Si/SiO_2_/Nb (${t}_{Nb}$)/CoFeB (0.9)/MgO (1)/Ta (2) (in nm), where ${t}_{Nb}$ = 3, 5, 7, 9, and 15 nm to obtain the SOT efficiency of Nb. Also, Si/SiO_2_/Nb (${t}_{Nb}$)/CoFeB (2)/MgO (1)/Ta (2) (in nm), where ${t}_{Nb}$ = 1, 2, 3, 4, 5, 7, 9, and 15 nm, films were fabricated to evaluate the thickness dependence of ${\xi }_{DL}$. Two other series of films with differing NM layers were placed under the Nb layer: Ta (3)/Nb (${t}_{Nb}$)/CoFeB (0.9)/MgO (1)/Ta (2) (Ta/Nb series) and Pt (3)/Nb (${t}_{Nb}$)/CoFeB (0.9)/MgO (1)/Ta (2) (Pt/Nb series). The thickness of the Nb layer varied from 3 to 15 nm for the Ta/Nb series and from 0 to 12 nm for the Pt/Nb series. All samples were post-annealed at 300 °C for 1 h at a magnetic field of 6 kOe applied perpendicularly to the film plane under a 10^−6^ Torr base pressure.

### Measurements

The magnetic properties of films were measured using a VSM (Microsense EV9) under two magnetic field directions, in-plane and perpendicular to the film plane. To measure the SOT, we patterned films that exhibited PMA into Hall bar structures that were 5 μm in width and 35 μm in length using photolithography and lift-off. The electrode consisting of Ti (10)/Au (100) (in nm) was evaporated using an e-beam. The Hall bars were wire-bonded and placed on the stage with motors. These motors rotated the device along the polar (θ) and azimuthal (φ) angles and the external magnetic field varied up to 18 kOe. We used a 1–3 mA alternating current (AC) during the measurement, with a fixed frequency of 13.7.

The electrical resistivity and current-induced SOT switching were measured using a 4-point probe station (MSTECH M7VC). We patterned the device using ion milling to fabricate a dot-shaped structure of the CoFeB/MgO/Ta layers with a 4 μm diameter to measure the SOT switching. We applied current with a 10 μs pulse width and an external magnetic field parallel to the current direction during measurement.

The microstructure and atomic distribution of samples were measured using TEM (TitanTM 80–300) at the Korea Institute of Science and Technology. The films' crystal structure was determined using XRD (PANalytical, X’Pert ProMPD) with a Cu Kα radiation source (λ = 1.5406 Å) at the Korea Basic Science Institute Seoul Western Center. The surface roughness of films was measured using AFM (Asylum Research MFP-3D) with SSS-SEIHR-20 cantilevers (SuperSharpSilicon, resonant frequency: 130 kHz, force constant: 15 N/m). We scanned 5 μm × 5 μm areas of each sample and operated imaging in non-contact mode at a scan frequency of 0.5 Hz.

## Supplementary Information

Supplementary Information.

## Data Availability

The data that support the finding of this study are available from the corresponding author on reasonable request.
