# Supplementary material for: Spin–orbit torques in normal metal/Nb/ferromagnet heterostructures
Source: Sci Rep. 2021 Oct 26;11:21081. doi: 10.1038/s41598-021-99745-4 (PMC8548299; doi:10.1038/s41598-021-99745-4)
Supplement: Supplementary file 1 — Supplementary Information. [file 41598_2021_99745_MOESM1_ESM.pdf]

Supplementary Information

Spin-orbit torques in normal metal/Nb/ferromagnet heterostructures

Min Hyeok Lee[1,4], Gyungchoon Go[2,4], Yong Jin Kim[1], In Ho Cha[1], Gyu Won Kim[1], Taehyun Kim[1], Kyung-Jin Lee[1,2,3], and Young Keun Kim[1*]

[1]Department of Materials Science and Engineering, Korea University, Seoul 02841, Republic of Korea.

[2]Department of Physics, Korea Advanced Institute of Science and Technology, Daejeon 34141, Republic of Korea.

[3]KU-KIST Graduate School of Converging Science and Technology, Korea University, Seoul 02841, Korea

[4]These authors contributed equally: Min Hyeok Lee, Gyungchoon Go.

[*]E-mail: ykim97@korea.ac.kr

Supplementary Note 1. Perpendicular magnetic anisotropy of Nb/CoFeB/MgO/Ta and NM/Nb/CoFeB/MgO/Ta

The magnetic hysteresis loops of Nb (t_{Nb})/CoFeB (0.9)/MgO (1)/Ta (2) (in nm) films with varying Nb thicknesses of 3, 5, 7, 9, and 15 nm were measured using VSM, as shown in Fig. S1. Magnetization (M) was normalized by dividing it by the saturation magnetization (M_s) of each film. The film with a 3 nm Nb layer exhibited in-plane magnetic anisotropy. However, all other films exhibited well-developed PMA regardless of t_{Nb}. The magnetic hysteresis loops of Ta (or Pt) (3)/Nb (t_{Nb})/CoFeB (0.9)/MgO (1)/Ta (2) (in nm) films were also measured. Note that t_{Nb} was varied as 3, 5, 7, 9, and 15 nm for the Ta/Nb series and as 0, 1, 2, 3, 4, 5, 7, 9 and 12 nm for the Pt/Nb series. The magnetization (M) was normalized by dividing it by the saturation magnetization (M_s) of each film. Except for the film with t_{Nb} = 3 nm, all films in the Ta/Nb series exhibited well-developed PMA, regardless of the t_{Nb}. In the Pt/Nb series, the PMA was obtained when Nb with a thickness of only 1 nm was inserted.

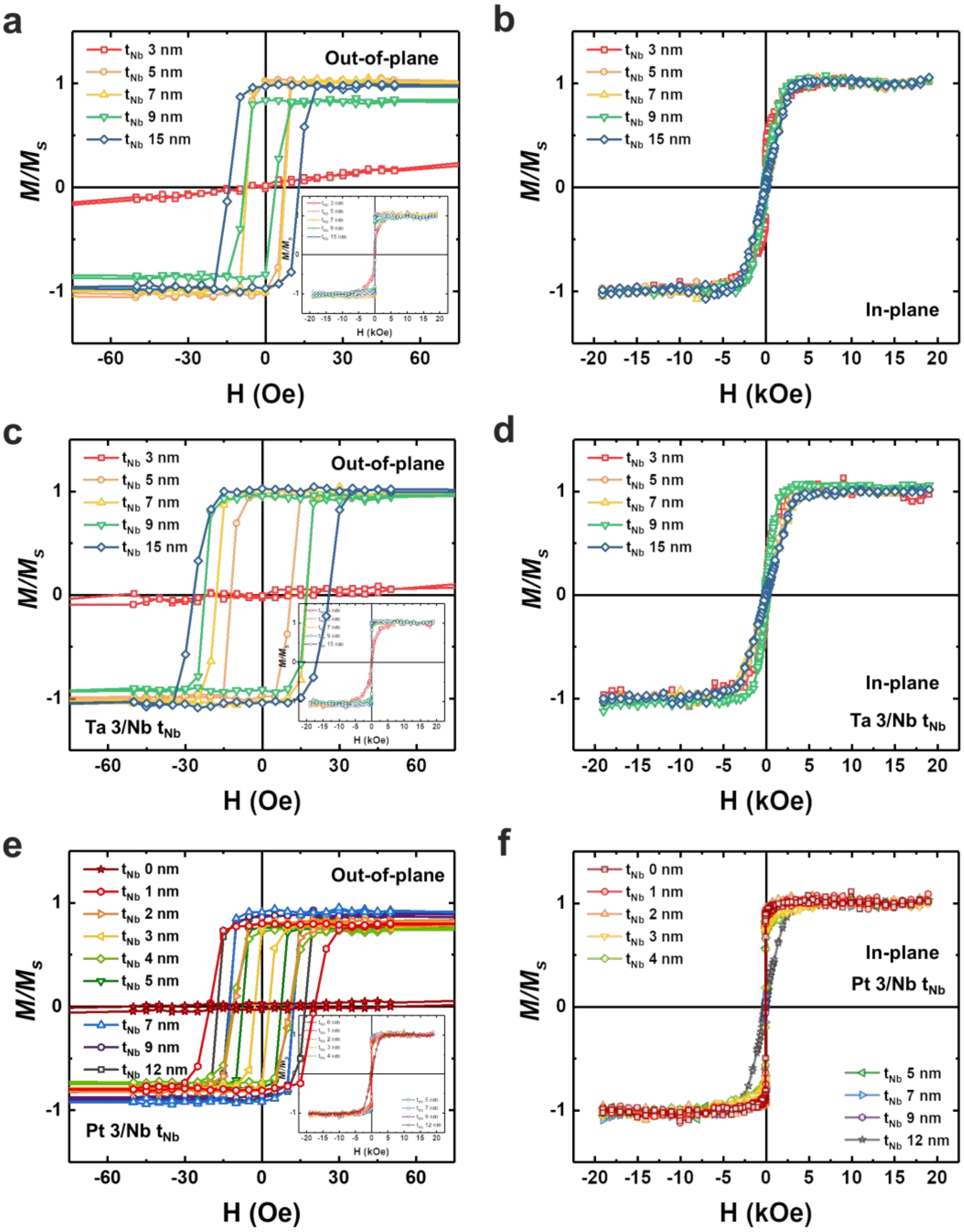

Fig. S1 Normalized magnetization of Nb/CoFeB/MgO/Ta and NM/Nb/CoFeB/MgO/Ta. a, b, out-of-plane (**a**) and in-plane (**b**) hysteresis loops for the 300 °C-annealed Nb (t_{Nb})/CoFeB 0.9/MgO 1/Ta 2 (in nm) films. **c, d,** out-of-plane (**c**) and in-plane (**d**) hysteresis loops for the 300 °C-annealed Ta 3/Nb (t_{Nb})/CoFeB 0.9/MgO 1/Ta 2 (in nm) films. **e, f,** show normalized out-of-plane (**e**) and in-plane (**f**) loops for the Pt 3/Nb (t_{Nb})/CoFeB 0.9/MgO 1/Ta 2 (in nm) films, respectively. In-set in the (**a**), (**c**), and (**e**) shows the out-of-plane full loops.

Supplementary Note 2. The 1st and 2nd harmonic curves for Nb based SOT structure

We measured all PMA films for their SOT efficiency using harmonics measurement. We induced the external magnetic field varying up to 18 kOe and used a 1–3 mA alternating current with a fixed frequency of 13.7 Hz. Fig. S2a and S2b show the 1st and 2nd harmonic signals of Nb (t_{Nb})/CoFeB (0.9)/MgO (1)/Ta (2), and S2c, S2d show the signals of Ta (3)/Nb (t_{Nb})/CoFeB (0.9)/MgO (1)/Ta (2), respectively. Those of Pt (3)/Nb (t_{Nb})/CoFeB (1.1)/MgO (1)/Ta (2) (in nm) structures are shown as Fig. S2e and S2f.

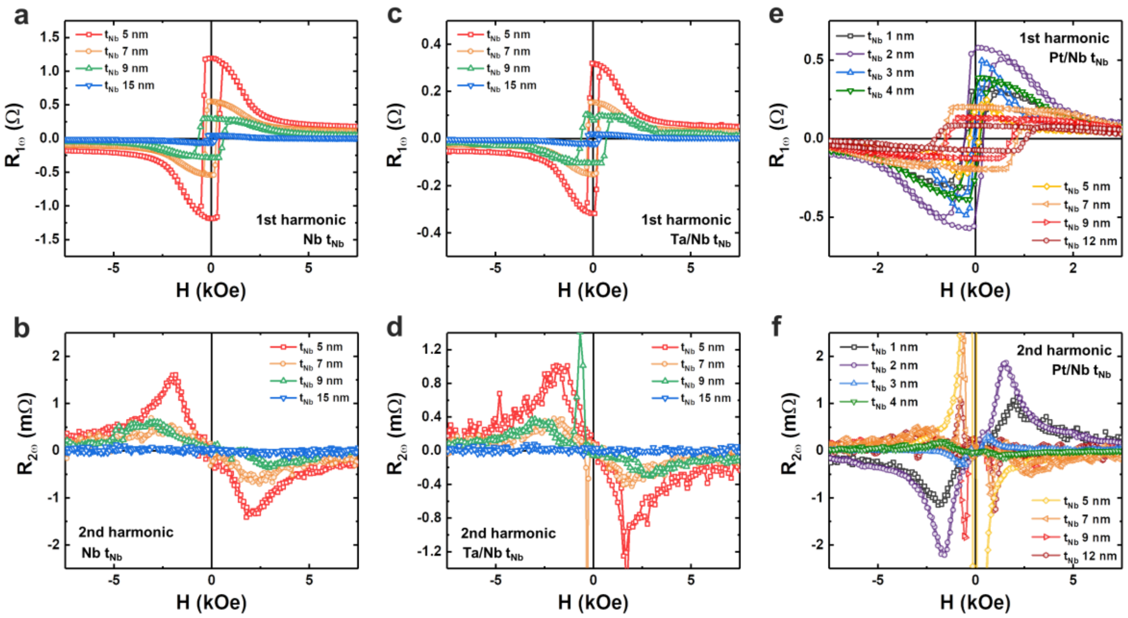

Fig. S2 Harmonics signals of Nb/CoFeB/MgO/Ta series. a, b, 1st harmonic **(a)** and 2nd harmonic **(b)** signals for Nb (t_{Nb})/CoFeB 0.9/MgO 1/Ta 2 films. **c, d,** 1st harmonic **(c)** and 2nd harmonic **(d)** signals for Ta (3)/Nb (t_{Nb})/CoFeB 0.9/MgO 1/Ta 2 films. **e, f,** 1st harmonic **(e)** and 2nd harmonic **(f)** signals for Pt (3)/Nb (t_{Nb})/CoFeB 1.1/MgO 1/Ta 2 films.

Supplementary Note 3. SOT measurement of the films with in-plane magnetic anisotropy

We patterned films into a Hall bar structure with a 5 μm width and 35 μm length using the photolithography and lift-off method to measure the spin Hall properties. The electrode consists of Ti (10)/Au (100) (in nm) was evaporated by e-beam. The Hall bars were wire-bonded and placed on the stage with motors that rotate the device in both angles; polar (θ) and azimuthal (φ) angles. The external magnetic field induced to the device varies up to 18 kOe. We used 1-3 mA of amplitude a.c current during the measurement, and its frequency was fixed as 13.7 Hz.

The magnetization of the films stays at an equilibrium position just before the inducing current. When current flows through the device, the magnetization starts to oscillate due to the effective field exerted by the spin current. We can obtain the spin Hall properties that reflect this oscillation from the θ and φ angle dependence between the measured Hall resistance and the magnetization direction. The first ($R_{xy}^{1\omega}$) and second-harmonic Hall resistance ($R_{xy}^{2\omega}$) is expressed as follows:

$$R_{xy}^{1\omega} = R_{\mathrm{AHE}} \cos\theta + R_{\mathrm{PHE}} \sin^2\theta \sin 2\varphi. \quad (1)$$

$$R_{xy}^{2\omega} = [R_{\mathrm{AHE}} - 2R_{\mathrm{PHE}} \cos\theta \sin 2\varphi] \frac{d\cos\theta}{d\mathbf{B}_I} \cdot \mathbf{B}_I + R_{\mathrm{PHE}} \sin^2\theta \frac{d\sin 2\varphi}{d\mathbf{B}_I} \cdot \mathbf{B}_I + R_{\nabla T} \sin\theta \cos\varphi, \quad (2)$$

Here, R_{AHE} and R_{PHE} are the anomalous Hall and planar Hall effect resistance, respectively. The second-harmonic Hall resistance ($R_{xy}^{2\omega}$) contains the degree of the magnetization oscillations in terms of the total effective field $\mathbf{B}_I = \mathbf{B}_{DL} + \mathbf{B}_{FL} + \mathbf{B}_{Oe}$. $\mathbf{B}_{DL}$ represents a SOT induced damping-like field, $\mathbf{B}_{FL}$ is a field-like field, and $\mathbf{B}_{Oe}$ is an electrical current induced-Oersted field. The Hall resistance occurred from a thermo-electric process expressed as $R_{\nabla T}$. In specific measurement geometry, $\theta = \pi/2$, we can get the simplified expression of second-harmonic Hall resistance.

$$R_{xy}^{2w} = \left[\left(R_{AHE}\frac{B_{DL}}{B_{eff}} + R_{\nabla T}\right)\cos\varphi + 2R_{PHE}(2\cos^3\varphi - \cos\varphi)\frac{B_{FL}+B_{Oe}}{B_{ext}}\right], \qquad (3)$$

where B_{eff} and B_{ext} are the effective anisotropy field and the applied external field, respectively[S1, S2]. When an applied external field is large enough to regard the direction of magnetization as parallel to that of B_{ext}, we can assume the contribution of thermo-electric Hall resistance is constant. Also, we can vanish the contribution of the $B_{FL} + B_{Oe}$ by rotating azimuthal angle into $\varphi = \pi/4$. In this case, we can separate the effect of B_{DL} with other effective fields. By measuring a relation between Hall resistance and azimuthal angle, the effect of $B_{FL} + B_{Oe}$ also could be obtained.

Supplementary Note 4. The resistivity of Nb films in (NM)/Nb/CoFeB/MgO structures

We measured ρ_{xx}^{Nb} in three systems: (i) Nb/CoFeB/MgO/Ta, (ii) Ta/Nb/CoFeB/MgO/Ta, and (iii) Pt/CoFeB/MgO/Ta. We fabricated two reference films consisting of Ta (3) or Pt (3)/CoFeB (0.9)/MgO (1)/Ta (2) to obtain ρ_{xx}^{Nb} in a parallel circuit model. The ρ_{xx}^{Nb} values corresponding to each thickness were calculated by deducting the ρ_{xx} of each reference film from the resistivity of the entire film. Following the introduction of the Ta seed layer, there is a reduction in ρ_{xx}^{Nb}, as shown in Fig. S3. In these systems, film thickness and ρ_{xx}^{Nb} were inversely proportional to each other. However, such a relationship between these parameters was not observed in the Pt/Nb series; this means that the Nb films above the Pt layer are of low quality.

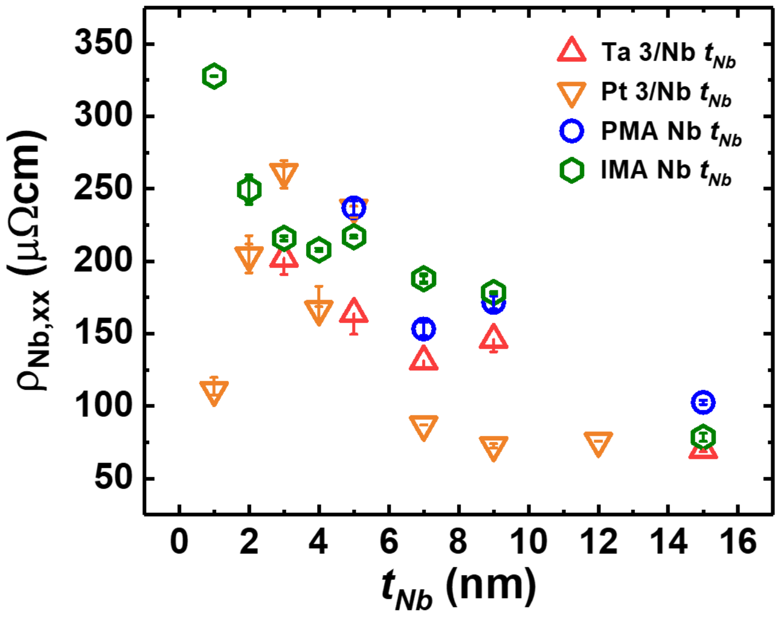

Fig. S3 Resistivity of Nb layer as a function of t_{Nb}. Values for the Nb t_{Nb}/CoFeB 0.9/MgO 1/Ta 2, Nb t_{Nb}/CoFeB 2/MgO 1/Ta 2, Ta 3/Nb t_{Nb}/CoFeB 0.9/MgO 1/Ta 2, and Pt 3/Nb t_{Nb}/CoFeB 1.1/MgO 1/Ta 2 (in nm) films.

Supplementary Note 5. Drift-diffusion model in the FM/NM1/NM2 structure

Here, we derived the analytical expression of the SOTs from the spin Hall effects in the NM1 and NM2 layers. A schematic of the NM1/FM and NM2/NM1/FM structures is provided in Fig. S4.

The perpendicular spin current densities in FM and NM are given by

$$\mathbf{j}_{s,z}^{F} = -\frac{\sigma_F}{2e}\partial_z \mu_s^F \mathbf{M} \quad \text{and} \quad j_{z,y}^{s,a} = -\sigma_{SH}^{a} E_x - \frac{\sigma^a}{2e}\partial_z \mu_{s,y}^{a}, \tag{S1}$$

where σ and σ_{SH} are the longitudinal and spin Hall conductivities, respectively, and μ_s is the spin accumulation. The subscript F denotes the FM layer, and the superscript *a* (= 1,2) differentiates the NM layers (NM1 and NM2). The spin accumulations satisfy the diffusion equation. Thus, we have

$$\mu_s^F = A^F e^{z/\lambda_{sf}^F} + B^F e^{-z/\lambda_{sf}^F}, \qquad \mu_{s,i}^{N,a} = A_i^a e^{z/\lambda_{sf}^a} + B_i^a e^{-z/\lambda_{sf}^a}, \tag{S2}$$

where λ_{sf}'s are the spin diffusion lengths, and coefficients *A* and *B* are determined by boundary conditions. The boundary conditions at the NM1/FM interface are given by[S3, S4]

$$j_z(0^-) = -(G_\uparrow + G_\downarrow)\left(\frac{\Delta\mu}{e}\right) - (G_\uparrow - G_\downarrow)\left(\mathbf{m}\cdot\frac{\Delta\boldsymbol{\mu}_s}{e}\right),$$

$$\mathbf{j}_{z,T}^{s}(0^-) = \frac{1}{2e}\left[\mathrm{Re}(G_{\uparrow\downarrow})(2\Delta\boldsymbol{\mu}_s \times \mathbf{m}) \times \mathbf{m} + \mathrm{Im}(G_{\uparrow\downarrow})(2\Delta\boldsymbol{\mu}_s \times \mathbf{m})\right],$$

$$j_{z,L}^{s}(0^-) = -\frac{1}{2e}\left[(G_\uparrow + G_\downarrow)(\mathbf{m}\cdot\Delta\boldsymbol{\mu}_s) + (G_\uparrow - G_\downarrow)\Delta\mu\right], \tag{S3}$$

where $\Delta\mu(\Delta\boldsymbol{\mu}_s)$ is the chemical potential (spin accumulation) drop over the NM1/FM interface; that is, $\Delta\boldsymbol{\mu}_s = \boldsymbol{\mu}_s^F(0^+) - \boldsymbol{\mu}_s^1(0^-)$. The additional boundary conditions were determined by the sample structure. For the NM2/NM1/FM structure, the additional boundary conditions were

$$j_z = 0, \quad \mathbf{j}_s^F(t_F) = \mathbf{j}_s^1(-t_{N1} - t_{N2}) = 0,$$

$$\boldsymbol{\mu}_s^1(-t_{N1}) = \boldsymbol{\mu}_s^2(-t_{N1}), \qquad \mathbf{j}_s^1(-t_{N1}) = \mathbf{j}_s^2(-t_{N1}). \tag{S4}$$

By solving Eqs. (S1)–(S4) for perpendicular magnetization ($\mathbf{m} = \mathbf{z}$), we obtained:

$$\frac{j^s_{z,x}(0^-)}{E_x} = \left(\sigma_{SH,1}C^1_{SH} + \sigma_{SH,2}C^2_{SH}\right)G_i\left(\lambda^2_{sf}\sigma_1\mathrm{th}_1 + \lambda^1_{sf}\sigma_2\mathrm{th}_2\right)D,$$

$$\frac{j^s_{z,y}(0^-)}{E_x} = -\left(\sigma_{SH,1}C^1_{SH} + \sigma_{SH,2}C^2_{SH}\right) \times$$

$$\left[\lambda^2_{sf}\sigma_1(|G|^2 + G_r\mathrm{th}_1) + \lambda^1_{sf}\sigma_2(|G|^2\mathrm{th}_1 + G_r)\mathrm{th}_2\right]D, \tag{S5}$$

where

$$C^1_{SH} = \left[\lambda^2_{sf}\sigma_1(1 - \mathrm{sh}_1) + \lambda^1_{sf}\sigma_2\mathrm{th}_1\mathrm{th}_2\right], \qquad C^2_{SH} = \lambda^2_{sf}\sigma_1\mathrm{sh}_1(1 - \mathrm{sh}_2),$$

$$D = \frac{1}{G_i^2\left(\lambda^2_{sf}\sigma_1 + \lambda^1_{sf}\sigma_2\mathrm{th}_1\mathrm{th}_2\right)^2 + \left[\lambda^2_{sf}\sigma_1(G_r + \mathrm{th}_{N1}) + \lambda^1_{sf}\sigma_2(1 + G_r\mathrm{th}_1)\mathrm{th}_2\right]^2},$$

and $G_r = \frac{2\lambda^1_{sf}}{\sigma_1}\mathrm{Re}G$ and $G_i = \frac{2\lambda^1_{sf}}{\sigma_1}\mathrm{Im}G$ are the real and imaginary components of the dimensionless mixing conductance, respectively. Here we use the abbreviations $\mathrm{th}_a = \tanh(t_a/\lambda^a_{sf})$ and $\mathrm{sh}_a = \mathrm{sech}\left(\frac{t_a}{\lambda^a_{sf}}\right)$. Note that taking the limit $t_{N2} \to 0$, the spin current expression in Eq (S5) becomes equivalent to that of the previous result of the FM/NM bilayer structure[S5].

Then, let us consider the effect of the interfacial spin current. For simplicity, we assume that the additional spin current is generated only at NM1/FM interface (we ignore the additional spin currents at other interfaces). In the drift-diffusion approach, the interface generated spin current modifies NM1/FM boundary conditions as follows[27, S6-S7]

$$\mathbf{j}^s_{z,T}(0^-) = \frac{1}{2e}\left[\mathrm{Re}(G_{\uparrow\downarrow})(2\Delta\boldsymbol{\mu}^s \times \mathbf{m}) \times \mathbf{m} + \mathrm{Im}(G_{\uparrow\downarrow})(2\Delta\boldsymbol{\mu}^s \times \mathbf{m})\right] + \mathbf{j}^{ISOC}_{s,T}(0^-),$$

$$j^s_{z,L}(0^-) = -\frac{1}{2e}\left[(G_\uparrow + G_\downarrow)(\mathbf{m}\cdot\Delta\boldsymbol{\mu}^s) + (G_\uparrow - G_\downarrow)\Delta\mu\right] + j^{ISOC}_{s,L}(0^-), \tag{S6}$$

where $\mathbf{j}^{ISOC}_s(0^-) = j^s_f\,\mathbf{y} + j^s_p(\mathbf{m} \times \mathbf{y})$. For perpendicular magnetization ($\mathbf{m} = \mathbf{z}$), the additional component of spin current solutions are

$$\frac{j_{z,x}^{s,ISOC}(0^-)}{E_x} = \left[\sigma_f G_i C_{ISOC}^1 - \sigma_p C_{ISOC}^2\right] D,$$

$$\frac{j_{z,y}^{s,ISOC}(0^-)}{E_x} = \left[\sigma_f C_{ISOC}^2 + \sigma_p G_i C_{ISOC}^1\right] D, \qquad (S7)$$

where

$$C_{ISOC}^1 = \left(\lambda_{sf}^2 \sigma_1 \mathrm{th}_1 + \lambda_{sf}^1 \sigma_2 \mathrm{th}_2\right)\left(\lambda_{sf}^2 \sigma_1 + \lambda_{sf}^1 \sigma_2 \mathrm{th}_1 \mathrm{th}_2\right),$$

$$C_{ISOC}^2 = \left(\lambda_{sf}^2 \sigma_1 \mathrm{th}_1 + \lambda_{sf}^1 \sigma_2 \mathrm{th}_2\right)\left[\lambda_{sf}^2 \sigma_1 (G_r + \mathrm{th}_1) + \lambda_{sf}^1 \sigma_2 (1 + G_r \mathrm{th}_1)\mathrm{th}_2\right].$$

From the spin current at the NM1/FM interface, we obtain the spin–orbit torque on the FM layer:

$$\boldsymbol{\tau} = \frac{\hbar}{2e}\frac{\gamma}{M_s t_F}\mathbf{j}_{z,T}^{s}(0^-) = \gamma[B_{DL}\mathbf{m} \times (\mathbf{m} \times \mathbf{y}) + B_{FL}\mathbf{m} \times \mathbf{y}]. \qquad (S8)$$

The expressions for the SOT efficiencies are described as:

$$\xi_{DL/FL} = \frac{2e}{\hbar}\frac{M_s t_F B_{DL/FL}}{j_c}. \qquad (S9)$$

Therefore, we have

$$\xi_{DL} = -\frac{j_{z,y}^{s}(0^-)}{j_c}, \qquad \xi_{FL} = -\frac{j_{z,x}^{s}(0^-)}{j_c}. \qquad (S10)$$

Note that j_c is the charge current density in the NM1/NM2 layer, which is obtained by the parallel circuit model

$$j_c = j_1 \frac{t_{N1}}{t_{N1} + t_{N2}} + j_2 \frac{t_{N2}}{t_{N1} + t_{N2}}. \qquad (S11)$$

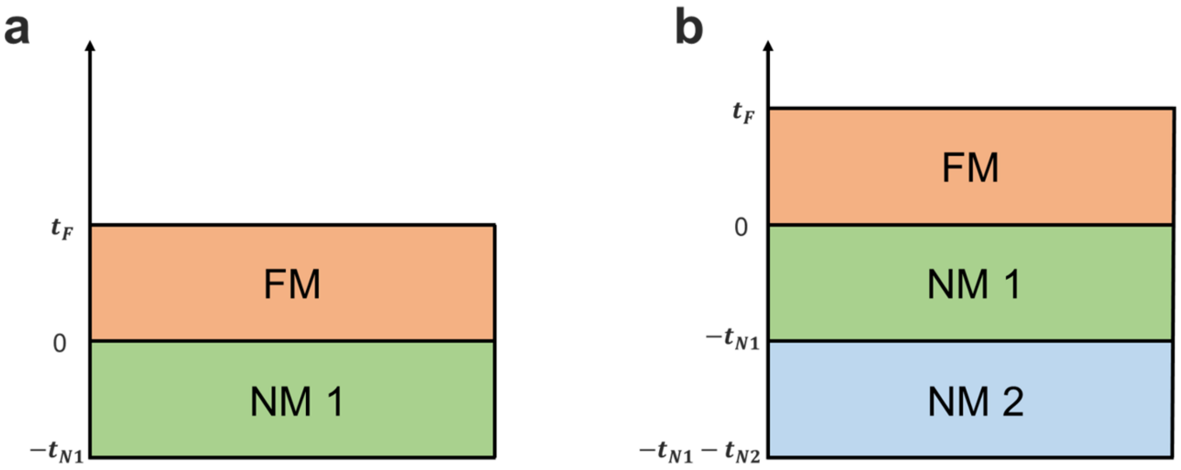

Fig. S4 Schematic structural design for theoretical calculation. a, NM1/FM and **b,** NM2/NM1/FM structures.

Supplementary Note 6. Spin diffusion length considering spin current generation at the normal metal/ferromagnet interface

We fitted the data using an extended spin diffusion model containing an interfacial source of spin current. We assumed that spin current is generated only at Nb/CoFeB interface for simplicity. Therefore, two conductivity parameters corresponding with spin-filtering and spin-precession were additionally considered for the Nb/CoFeB boundary conditions modified by interface generated spin current[27] (see Supplementary Note 5). $\lambda_{s,Nb}^{eff}$ = 3.64 ± 0.72 nm was obtained for the Nb/CoFeB bilayer system, as shown in Fig. S5a. For reducing the ambiguity of the tri-layer system fitting, we assumed that the parameters describing interfacial conditions were the same as those of the Nb/CoFeB bilayer system. Fig. S5b and S5c showed that the extracted values were $\lambda_{s,Nb}^{eff}$ = 8.61 ± 1.83 and $\lambda_{s,Nb}^{eff}$ = 4.48 ± 1.00 nm in Ta/Nb and Pt/Nb series, respectively. $\lambda_{s,Nb}^{eff}$ extracted by using an extended model slightly increased compared to those when the conventional bulk model was applied, but the enhancement was negligible in all cases.

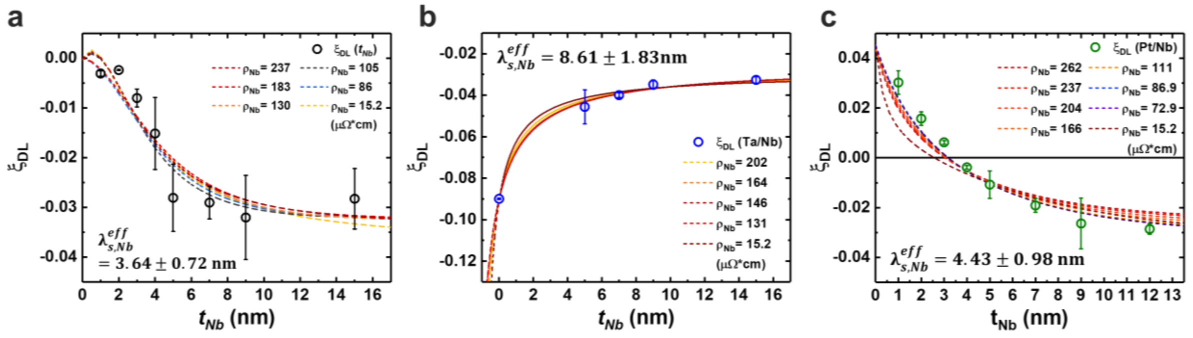

Fig. S5 Extraction of the spin diffusion length considering Nb/CoFeB interface. a, b, c ξ_{DL} as a function of t_{Nb} in the Nb/CoFeB bilayer and Ta (Pt)/Nb/CoFeB tri-layers series, respectively. Each dotted line shows the fitting using the drift-diffusion equation considering spin current generated at the Nb/CoFeB interface with different resistivities of Nb.

Table S1 Model-dependent spin diffusion lengths of Nb (in nm).

$\lambda_{s,Nb}^{eff}$	Nb/CoFeB	Ta/Nb/CoFeB	Pt/Nb/CoFeB
Conventional Bulk Model	2.90 ± 0.52	8.17 ± 1.68	4.40 ± 0.96
Bulk + Interface Spin Current Model	3.64 ± 0.72	8.61 ± 1.83	4.43 ± 0.98

Supplementary Note 7. NM 1/NM 2 interface analysis

To characterize the structural properties of these systems, we deposited an NM/Nb bi-layer and then conducted a TEM analysis. Fig. S6a and S6b show the side-views of Ta 3/Nb 5 nm and Pt 3/Nb 5 nm, respectively. Note that annealing at 300 °C for 1 h was conducted for both samples. The situations at the NM layer and the NM/Nb interface were completely different. The 3 nm Ta layer maintains an amorphous structure, despite heat treatment. It is well-known that films deposited over the amorphous buffer layer show well-defined interface quality and crystallinity[S8-S10], as demonstrated by Fig. S6a. When very thin layers of Pt were grown on a substrate lacking a buffer layer, Pt exhibited an amorphous structure. However, in the Pt/Nb structure, the Pt layer after annealing at 300 °C featured polycrystallinity, as shown in Fig. S6b. Besides, we observed a low-quality interface that appears to have formed during the crystallization process. To verify this in duplicate, a TEM energy dispersive spectroscopy (EDS) analysis was also conducted. Each inset of Fig. S6a and S6b depicts the atomic ratio between NM and Nb for the regions represented as Regions 1 and 2, respectively. Nb atoms were almost absent in the Ta region (~0.40%), whilst some Nb atoms diffused into the Pt region (~17.8%) for Pt/Nb. The atoms diffused into the NM layer, and the resulting interfaces inevitably affected the transport of current through scattering; the drift-diffusion model does not consider this.

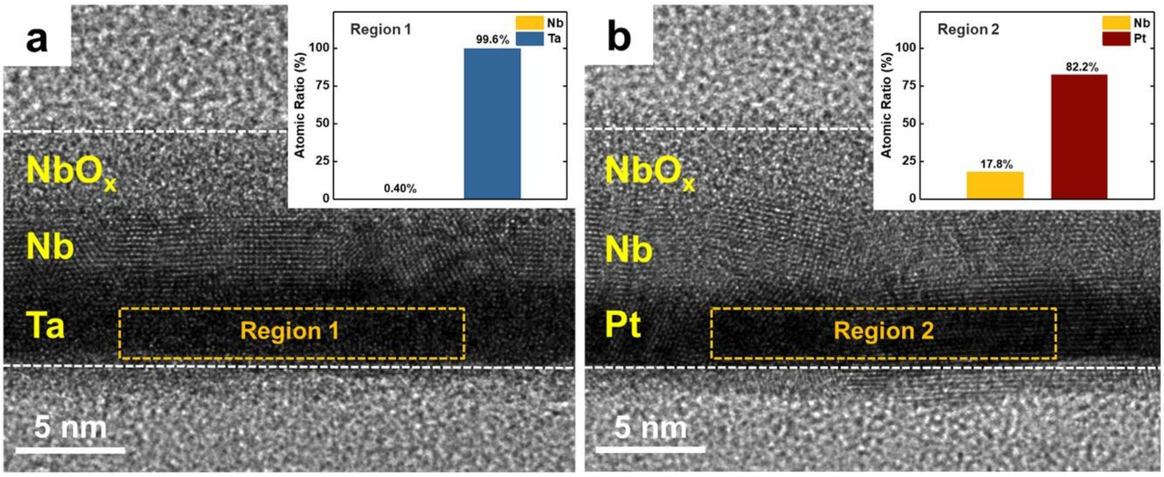

Fig. S6 Comparison of NM1/NM2 interface states based on material combinations. a, b, TEM analysis of Ta 3/Nb 5 nm **(a)** and Pt 3/Nb 5 nm films **(b)** following annealing at 300 °C for 1 h. The white dotted line shows the boundary of films. The inset of each figure shows the atomic ratio between Nb and Ta **(a)** and Pt **(b)** for the region indicated by the dark yellow dotted line.

Supplementary Note 8. Surface roughness of Nb thin films on different underlayers

We measured the roughness of Nb thin films using atomic force microscopy (AFM) to confirm the effect of the underlayer type on the interfacial roughness of Nb films. We scanned 5 $\mu m \times 5\mu m$ areas of each sample, and the root-mean-square (RMS) of the area was indicated as a data point. We indicated the error range using the standard deviation. The overall trend of roughness increases as t_{Nb} increases regardless of the type of underlayers, as shown in Fig. S7. The Nb thin films grown on Ta underlayer were less rough than the other series grown on SiO_2 and Pt underlayer. The films grown on SiO_2 and Pt showed higher roughness than the Ta series but a similar roughness level. Although the overall tendency was similar in all series, there were the difference in Nb roughness between Ta/Nb and Pt/Nb series that may induce different transport behavior at the Nb/CoFeB interface.

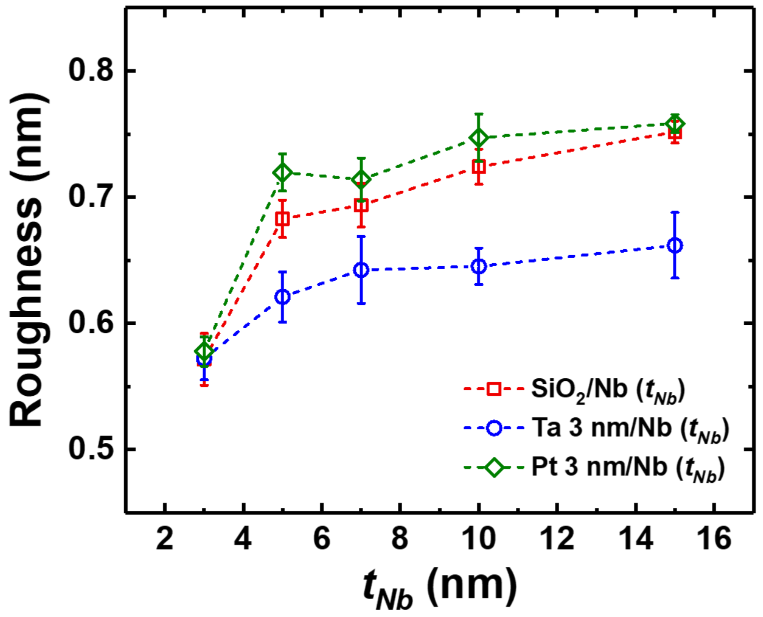

Fig. S7 AFM analysis of Nb thin films with the different buffer layers.

References

S1. Avci, C. O. *et al.*, Interplay of spin-orbit torque and thermoelectric effects in ferromagnet/normal-metal bilayers, *Phys. Rev. B* **90**, 224427 (2014).

S2. Avci, C. O., Beach, G. S. D. & Gambardella, P. Effects of transition metal spacers on spin-orbit torques, spin Hall magnetoresistance, and magnetic anisotropy of Pt/Co bilayers, *Phys. Rev. B* **100**, 235454 (2019)

S3. Brataas, A., Tserkovnyak, Y., Bauer, G. E. W. & Halperin, B. I. Spin battery operated by ferromagnetic resonance, *Phys. Rev. B* **66**, 060404(R) (2002).

S4. Polianski, M. L. & Brouwer, P. W. Current-Induced Transverse Spin-Wave Instability in a Thin Nanomagnet, *Phys. Rev. Lett*. **92**, 026602 (2004).

S5. Haney, P. M., Lee, H.-W., Lee, K.-J., Manchon, A. & Stiles, M. D., Current induced torques and interfacial spin-orbit coupling: Semiclassical modeling, *Phys. Rev. B* **87**, 174411 (2013).

S6. Amin, V. P. & Stiles, M. D., Spin transport at interface with spin-orbit coupling: Formalism. *Phys. Rev. B* **94**, 104419 (2016).

S7. Amin, V. P. & Stiles, M. D., Spin transport at interface with spin-orbit coupling: Phenomenology. *Phys. Rev. B* **94**, 104420 (2016).

S8. Kim, S., Lee, S., Kim, J., Kang, J. & Hong, J. Microstructures and perpendicular magnetic properties of Co/Pd multilayers on various metal/MgO seed-layers, *J. Appl. Phys*. **109**, 07B766 (2011).

S9. Takeuchi, T. *et al*., Crystallization of Amorphous CoFeB Ferromagnetic Layers in CoFeB/MgO/CoFeB Magnetic Tunnel Junctions, *Jpn. J. Appl. Phys*. **46**, L623 (2007).

S10. Peng, W., Keitel, O., Victora, R. H., Koparal, E. & Judy, J. H. Co/Pt Superlattices with Ultra-Thin Ta Seed Layer on NiFe Underlayer for Double-Layer Perpendicular Magnetic Recording Media, *IEEE Trans. Magn*. **36**, 2390 (2000).
